# Current issues in patient safety in surgery: a review

**DOI:** 10.1186/s13037-015-0067-4

**Published:** 2015-06-05

**Authors:** Fernando J. Kim, Rodrigo Donalisio da Silva, Diedra Gustafson, Leticia Nogueira, Timothy Harlin, David L. Paul

**Affiliations:** Division of Urology, Department of Surgery, Denver Health Hospital and Authority, University of Colorado School of Medicine, 777 Bannock St., Denver, CO 80204 USA; Administration, Denver Health Hospital and Authority, Denver, CO USA; Department of Business Information & Analytics, Daniels College of Business, University of Denver, Denver, CO USA

## Abstract

Current surgical safety guidelines and checklists are generic and are not specifically tailored to address patient issues and risk factors in surgical subspecialties. Patient safety in surgical subspecialties should be templated on general patient safety guidelines from other areas of medicine and mental health but include and develop specific processes dedicated for the care of the surgical patients. Safety redundant systems must be in place to decrease errors in surgery. Therefore, different surgical subspecialties should develop a specific curriculum in patient safety addressing training in academic centers and application of these guidelines in all practices. Clearly, redundant safety systems must be in place to decrease errors in surgery, in analogy to safety measures in other high-risk industries. Specific surgical subspecialties are encouraged to develop a specific patient safety curriculum that address training in academic centers and applicability to daily practice, with the goal of keeping our surgical patients safe in all disciplines. The present review article is designed to outline patient safety practices that should be adapted and followed to fit particular specialties.

## Introduction

More than 200 million surgeries are performed worldwide each year and recent reports reveal that adverse event rates for surgical conditions remain unacceptably high, despite multiple nationwide and global patient safety initiatives over the past decade [1, 2]. These include the ‘100,000 Lives Campaign’ (2005/2006) and subsequent ‘5 Million Lives Campaign’ (2007/2008) by the Institute for Healthcare Improvement (IHI), the ‘Surgical Care Improvement Project’ (2006) and ‘Universal Protocol’ (2009) by the Joint Commission, and the WHO ‘Safe Surgery Saves Lives’ campaign accompanied by the global implementation of the WHO surgical safety checklist (2009) [3–5]. Interestingly, adverse events resulting from surgical interventions are actually more frequently related to errors occurring before or after the procedure than by technical surgical mistakes during the operation. These include *(i)* breakdown in communication within and amongst the surgical team, care providers, patients, and their families; *(ii)* delay in diagnosis or failure to diagnose; and *(iii)* delay in treatment or failure to treat [6–8]. On a daily basis, surgeons must adjudicate challenges that reach far beyond pure technical aspects - the decision of initiating appropriate and timely surgical care weighed against the risk of providing delayed or negligent care by rather choosing observation and/or non-operative treatment. These specific characteristics should trigger surgical subspecialties to add their specific patient safety processes and guidelines to the existing global ones.

We reviewed the current issues in patient safety in surgery including: a) general guidelines i.e.; the World Health Organization (WHO) pre-operative check list, communication gaps between the surgeons and staff and/or patient, b) organizational processes to prevent errors (Reason’s Swiss cheese model) and miscommunication, culture of safety and conflict resolutions.

### General considerations

Despite changes in the health care system with new regulatory mandates and reimbursement issues, one constant concern is to ensure exceptional patient safety and care.

Patient care must be delivered safely by utilizing safety guidelines based on scientific evidence. Constant revision of processes and guidelines are in order to optimize patient experience and safety. To do so, patient safety systems should focus on building a culture of safety that encourages communication, trust, and honesty [9].

In this process it is pivotal to recognize that humans make errors [10]. Failures occur by choosing the inappropriate method of care or by poor execution of an appropriate method of care. Fortunately, errors can be minimized with proper training, effective communication, and a system of checks and balances. Continual education regarding patient safety not only helps health care professionals by inhibiting errors, but also extends to patient well-being. Concise communication with patients instills trust and strengthens patient-provider relationships. Establishing a medical system of checks and balances ensures that errors are more likely to be caught before they happen and that blame does not rest upon an individual.

**Fig. 1:**
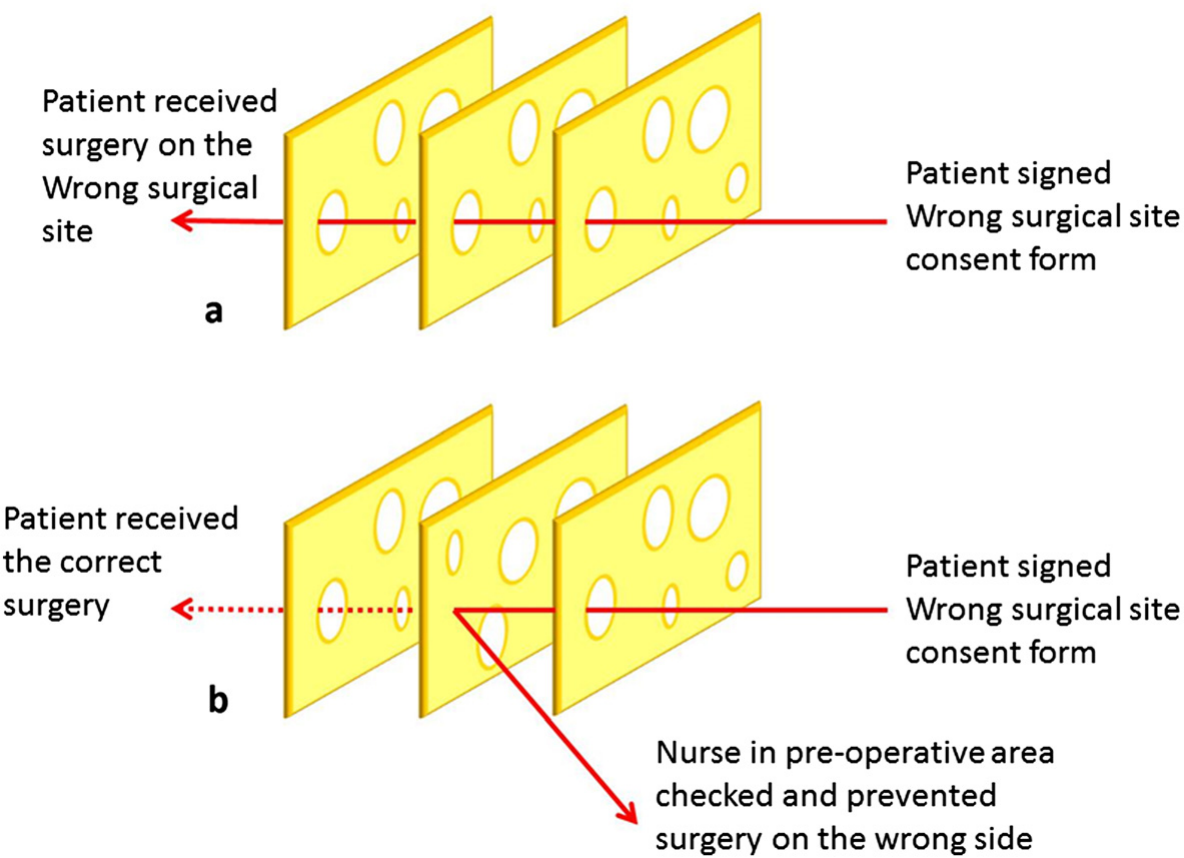
Swiss Cheese Model **a**) Accidents are aligned and layers of defense do not lie between, therefore accidents can occur. **b**) Accidents are not aligned and layers of defense do not lie between, therefore accidents are more difficult to occur

Errors are inevitable, but having a system in place to prevent them from occurring, and remedying them when they do occur, improves overall patient safety in the health care environment. Therefore, the “Swiss Cheese Model” originally formally propounded by Dante Orlandella and James T. Reason of the University of Manchester, what he referred to as system failure model [11]. Every step in a process has the potential for failure, to varying degrees. The ideal system is analogous to a stack of slices of Swiss cheese. Consider the holes to be opportunities for a process to fail, and each of the slices as “defensive layers” in the process. An error may allow a problem to pass through a hole in one layer, but in the next layer the holes are in different places, and the problem should be caught. Each layer would work as a defense against potential error impacting the outcome. The more number of defenses, the fewer and the smaller the holes, the more likely you are to catch and stop errors that may occur [11]. The Swiss cheese model of accident causation illustrates that if hazards and accidents are aligned and layers of defense do not lie between, the flaws in each layer can allow the accident to occur (Fig. 1).

The healthcare system can learn from other industries that are also considered HRO. Grote et al. described how the absence of shared cultural norms and infrequent communication across the organization could compromise the organization’s safety [12].

The most logical process to improve patient safety in health care systems is proposed below:

1. Identify current issues regarding patient safety
2. Revise systems, education, and training to address known patient safety issues
3. Educate health care professionals about the importance of patient safety concepts. Establish a system of checks and balances to reduce medical errors. Ensure practical application of patient safety concepts (training)
4. Enhance patient interaction to reduce errors

Repeat the process to address errors that persist.

### 1-Identify current issues regarding patient safety

An initial assessment of the current patient safety guidelines is necessary to remedy issues within the system. Medical errors are inevitable in the health care profession, but by identifying causes and developing plans to minimize or eliminate them can help to establish an effective system that ensures patient safety. Some causes of errors in health care systems are [9]:

- Lack of continuous training and education
- Past tolerance of unsafe practice
- Lack of regulations/rules
- Gaps in communication among different healthcare providers
- Gaps in communication between healthcare providers and patients
- Unstable/unreliable systems
- Fear of admission of guilt/wrongdoing
- Human factors

### 2: Revise systems, education, and training to address known patient safety issues

Once the issues impeding patient safety have been identified, plans can be established to limit or eliminate them. One treatable factor is the “culture of blame” present in health care systems. Admitting wrongdoing is often avoided for fear of being penalized. Employees should welcome the learning opportunity that mistakes can provide. The system should be modified to encourage teamwork, improve accountability, and reduce individualized blame. There are two facets that should be addressed: a) process and b) culture of patient safety.

**Fig. 2:**
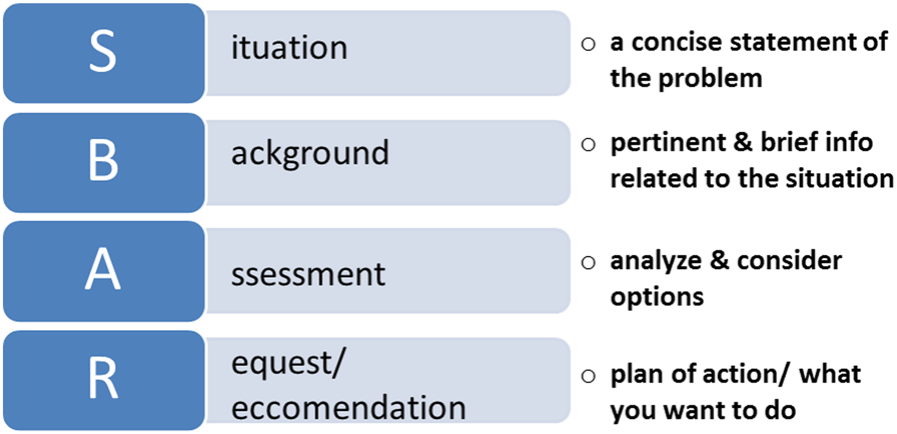
Mnemonic system to establish a structure of communication

- Process
  Employees benefit from clear rules and transparent processes. The World Health Organization (WHO) has a safety checklist that should be adapted into the current system [9]. It clearly addresses patient safety issues, like allergies, that can be overlooked and lead to severe consequences [9, 13].
  The Surgical Safety checklist includes three well-defined steps where the surgical team communicates and identifies possible risks for errors.
  Step 1: Before the induction of anesthesia - a nurse and the anesthesiologist will confirm the patient’s identity, site of surgery, procedure, and check the surgical consent form.
  Step 2: Before the skin incision - the nurse, anesthesiologist and the surgeon will confirm the role and names of the team members, reconfirm the patient’s name, verify the procedure, and check the incision site. The team will also confirm whether antibiotic prophylaxis was given within the last 60 min. Furthermore, the surgeons, anesthesiologist, and nursing team will identify anticipated critical events, i.e.; the length of the case, possible significant blood loss, patient-specific concerns, and equipment issues.
    Specifically for the urologists, this step will require that the display of essential imaging is verified, i.e.; Computerized Tomography (CT) scan for urolithiasis therapy, nephrectomy, etc.…
  Step 3: Before the patient leaves the operating room - the nurse, anesthesiologist, and surgeon will verbally confirm the name of the procedure, availability of adequate instrumentation, sponge and needle counts, specimen labelling (if applicable), issues with equipment, and key concerns for recovery and management of this patient.
- Culture of Patient Safety and Improving Communication among team members:
  Success in patient safety depends on optimal line of communication between surgeons, administrators and other healthcare providers to obtain and apply the necessary resources and improve means of communication and awareness.
  Ineffective team communication, especially in the operation room (OR), is a major root cause of these errors [14]. Mickan et al. described six characteristics of an effective team involving purpose, goals, leadership, communication, cohesion, and mutual respect. Incorporating these qualities into medical communities can minimize errors and improve patient safety [14].
  One effective tool used to help assess problems and resolve conflicts ion communication and other issues is SBAR (Situation, Background, Assessment, and Recommendation). SBAR is an effective and efficient way to communicate important information. SBAR offers a simple way to help standardize, set expectations, and establish structure of communication [15] (Fig. 2).
  **S**ituation: a concise statement of the problem
  **B**ackground: pertinent and brief information related to the situation
  **A**ssessment: analysis and considerations of options — what you found/think
  **R**ecommendation: action requested/recommended — what you want

SBAR allows all parties involved in the discussion to be on the same page, proactively giving the listener necessary data and recommendations to solve the problem. A similar commonly used protocol by physicians is the SOAP note (subjective, objective, assessment and plan). Both tools help to establish a culture of patient safety.

### 3: Training - Educate health care professionals about the importance of patient safety concepts. Establish a medical system of checks and balances to reduce medical errors. Ensure practical application of patient safety concepts

The next step to improve patient safety in health care systems is to apply the updated rules to the system. Health care professionals should be trained to encourage team work, “systems thinking”, honesty, and policy adherence. “Systems thinking” helps employees approach problem solving by seeing individual issues as parts of a whole [9]. If there is a checklist before each procedure, the staff needs to know how to accurately complete it and why it is important to do so. Every employee should be aware of their role in the health care process and alert to possible errors. When health care professionals work together and are properly trained, patient safety can substantially improve. Training may vary among medical care facilities and should be formatted to adhere to policies, regulations, and environments present within the system.

### 4: Enhance patient interaction to reduce errors

At this point in the process, implemented changes can be used during patient interaction. Employees should be following all rules and making sure they are communicating effectively with their patients and with each other. When errors occur, repeat the process.

### Institutional protocols and training

General requirements for emergent and elective care of patients must include screening exams and patient’s consent for care and surgery. In certain emergencies and life-threatening situations the caregivers may not have the ability to obtain proper authorization for care or surgery from the next of kin. In these rare situations, good communication between healthcare providers and others (administrators, social services and law enforcement), as well as effective use of technology (electronic medical record) is necessary to increase patient safety and decrease possible errors in the system (e.g., unknown co-morbities, allergies and past medical history) [16]. General guidelines regarding patient safety begin with verification of procedural steps such as patient identification, surgical site, positioning, and preparation [17].

Institutional protocols and proper training of personnel should be revised often and current for all steps of patient care including such things as radiation concerns during radiological imaging, environmental safety (sterilization, prevention and dissemination of infection, etc.…), and laboratory Services. The National Patient Safety Goals state that the patient should be identified by two or more methods, the test results should be returned promptly to the appropriate staff member, and proper sanitation guidelines outlined by an accredited organization should be followed [18].

## Specific considerations for surgical specialties

### Surgical procedures

Health-care workers should be trained to reduce misinformation or inconsistent information that can lead to errors, such as wrong-site surgery [19–21].

#### Scheduling the procedure

Office schedulers must carefully verify patient documentation before scheduling the procedure. All surgery requests must be in writing. No verbal requests by the medical staff should be accepted. An appropriate scheduling form reduces misunderstandings. Illegible handwriting, unapproved abbreviations, and cross-outs can be pitfalls if not clearly understood by office schedulers. Electronic medical records can improve the safety process, reducing misunderstandings and missing documents [21–23].

Verification of every pertinent document such as consent, history, physicals, and surgeon orders at time of scheduling is mandatory. If any inconsistency is found within the documentation during the process, office-schedulers should be instructed not to proceed to the next step without solving conflict or absence of information.

#### Pre-operative

The preoperative visit is another opportunity to identify and correct any inconsistencies or lack of information in the documentation regarding the surgical procedure. All documents should be checked during the visit and the patient should confirm identity, site of surgery, allergies, and other pertinent information if possible. All discrepancies must be corrected on all forms and documents prior to moving forward.

The informed consent must be received prior to the procedure and the patient must fully understand their procedure including things such as complications, additional procedures, placement of stents, and important alternative treatments that may be used in the present case [19, 20, 24, 25].

Marking the site of the procedure is critical in order to avoid wrong-site surgery. Preferentially, site marking should be performed with the patient’s involvement [26, 27]. The site must be marked by a licensed practitioner who is responsible for the procedure and will be present when the procedure is performed. The marks should be unambiguous and uniform within the institution and should be semi-permanent to be visible after skin preparation and draping [28].

In case marking the site is not possible due to technical or anatomical impediments (mucosal surfaces, minimal access procedures, endoscopic procedures, natural orifice procedures, etc.), the institution should have a written process to ensure that the correct site is operated on [29, 30]. Alternatively, radiopaque markers can be used in the procedures involving fluoroscopy [29, 31, 32].

Another important aspect of patient safety is the surgical material used during the procedure. Availability of all instruments and special materials (e.g., guide wires, laser fibers, scopes, stents, loops, prosthesis, etc.) should be verified prior to surgery and checked to ensure that they are the appropriate size for the patient [33].

#### Before starting the procedure

Full implementation of safety checklists in surgery has been linked to improved outcomes [9, 13, 34, 35]. The World Health Organization checklist is designed to identify a potential error before it results in harm to a patient. This checklist should be followed in the appropriate manner.

In a study by Russ S. et al., more than 40 % of cases had absent team members, and over 70 % of team members failed to pause and focus on the checks [13]. Performing a time-out and implementing a check list in the operating room does not mean that the patient is safe. Team members still have to adhere to the protocols and follow them with full attention. Surgical safety performance was better when surgeons led the procedure and all team members were present and paused [13]. The time-out must be documented at its completion. When multiple procedures are going to be performed on the same patient by different providers, the check list and time out should be performed for each procedure.

In the era of digital images, displaying the CT-scan, X-ray, and all other pertinent images during the procedure on an auxiliary monitor can improve patient safety [36, 37].

The consequences of positioning related injuries are preventable but can be profound and can result in morbidity and litigation [38]. Neurological, vascular, musculoskeletal, and pressure ulcers are the most common position related injuries in surgical patients [38, 39]. Neurological complications can be avoided by placing forearms in neutral position or slightly supinated to minimize pressure in the cubital tunnel [40]. Straps should be properly placed to maintain the correct limb position during the procedure even if the surgical table is moved. The patient’s head should be placed in a neutral position and the arm should not exceed abduction of more than 90° to prevent brachial plexus injury. Straps should not be too tight to avoid ischemia and compartmental syndrome. Padding under osseous prominences can help avoid pressure-related complications. Urologists must be careful to avoid possible compartment syndrome (limbs) when positioning patients for open, endoscopic, and laparoscopic surgeries [41–43].

#### Before discharge from the facility

Discharge planning has been shown to impact patient safety, patient outcomes, and can prevent readmissions and improve patient satisfaction [44–46]. Patient education is crucial when they are discharged home with catheters, stomas, stents, drains, or any other medical device that needs special care. Patient education can reduce complications and improve patient quality of life after surgical procedures [47–49]. Heath care workers must be aware that language barriers, socioeconomic status, and age can impact patient comprehension of instructions [49–51]. Written instruction must also be provided and follow-up visits should be scheduled prior to patient discharge from the facility.

### Laboratory exams, biopsies, and surgical pathology

Office-procedures such as biopsies (prostate, skin lesion, bladder), urine cultures, and blood samples are routine in a urology clinic. The large amount of patients, multiple samples from the same patient, lack of staff, and lack of continuous education and training of health care workers may increase medical errors. Approximately 1 % of general laboratory specimens are misidentified and can lead to serious harm for patients [52].

For patient safety, prevention is the goal and can be accomplished by implementing safety strategies. Health care workers responsible for specific tasks must be educated and motivated to perform those tasks with as few errors as possible [53]. Written policies and protocols detailing responsibilities must be implemented along with a strategic plan to detect errors when these responsibilities are not met. Successful completion of required tasks must be documented in order to move forward, especially in those tasks that are performed as a prerequisite to others [53].

To make the process as simple as possible, reduce the number of steps between collecting the samples and receiving the laboratory report. Redundancy checks must be encouraged in certain steps of the process in order to increase the chance of detecting mistakes before a therapeutic decision is made, especially when the decision is irrevocable and the potential damage caused by error cannot be undone.

Procedures that involve biopsy and tissue sampling a specimen may pass through the hands of more than twenty individuals in several workplaces until the final pathology report is given [54]. These handoffs significantly increase the risk of a mix-up and can lead to serious diagnostic errors. Mutual cooperation for supervision of clinicians, technicians, and administrative assistants is essential to prevent and detect errors. The most vulnerable steps of the biopsy process include labeling of the specimens, appropriate request forms, and accessioning of biopsy specimens [54].

**Fig. 3:**
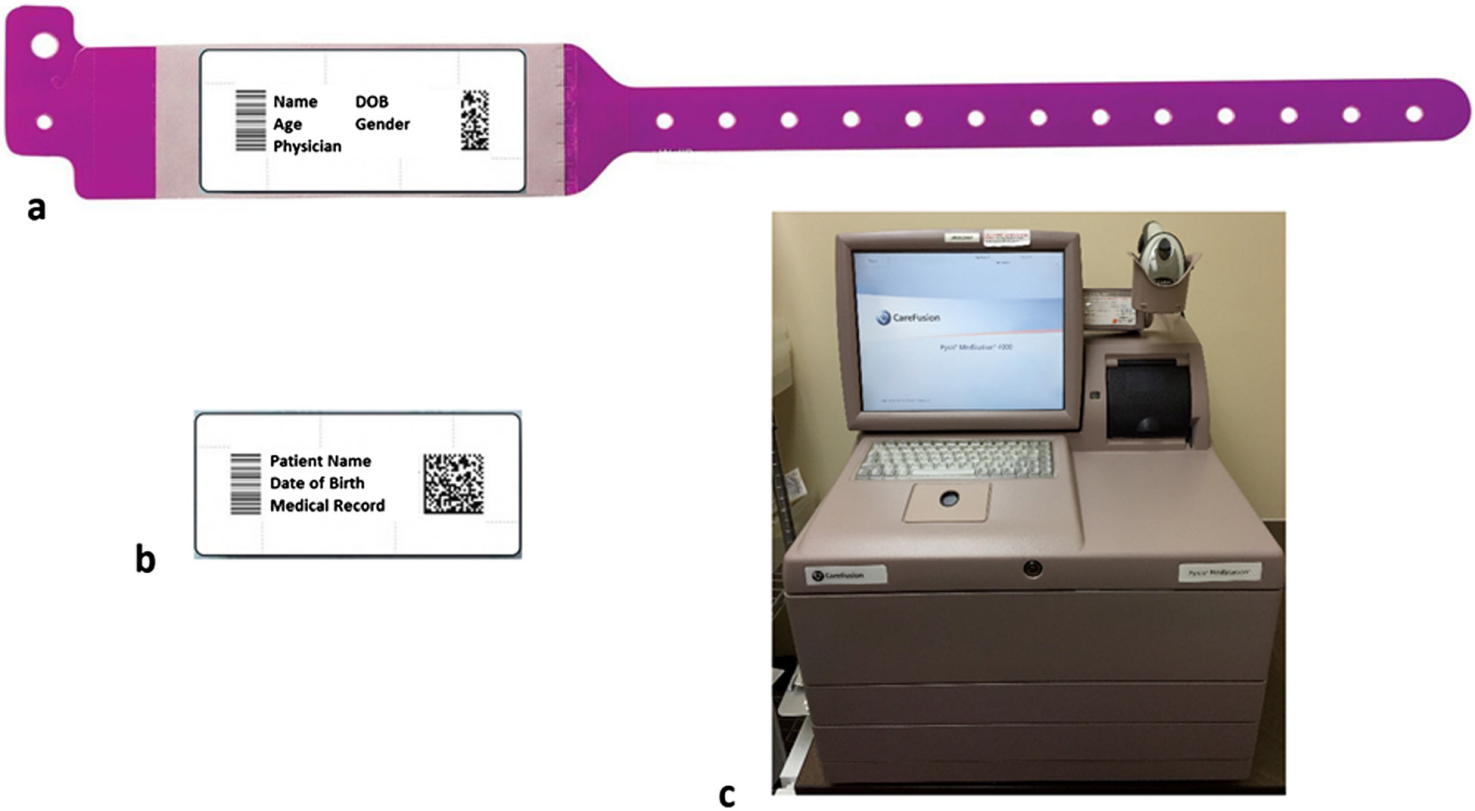
Automated systems for patient identification **a**) Wristband with patient identification and barcode **b**) Sticker with patient identification and barcode to be used with samples, charts, prescriptions, etc. **c**) Medication dispensary device, requires medical record number and barcode scan to dispense correct medication to correct patient

The use of information technology for data entry, automated systems for patient identification and specimen labeling, as well as two or more identifiers during sample collection are important steps to reduce misidentification [54, 55] (Fig. 3). If misidentification is detected, rejection then recollection is the most suitable approach to manage the specimen. DNA analysis to assist with correct identification can be used when recollection is not available [56].

### Medication safety

Medication safety can be improved by utilizing the five R’s: right drug, right route, right time, right dose, and right patient. Medication errors are barriers that prevent the right patient from receiving the right drug in the right dose at the right time through the right route of administration at any stage during medication use, with or without the occurrence of adverse drug events [57]. Medication errors represent the largest single cause of errors in the hospital setting in the United States, and are estimated to harm at least 1.5 million patients annually [57, 58].

In 2009, the government spent $30 billion in taxpayer subsidies toward the transition to digital medical records. Electronic medical records helped to decrease medication error and medication reconciliation by up to 50 % [59, 60]. Systems that use information technology, such as computerized physician order entry, automated dispensing, barcode medication administration, electronic medication reconciliation, and personal health records are vital in the prevention of medication errors [58]. Electronic medical records provide pharmacists with the ability to rapidly screen the medication regimens of hospitalized patients and deliver timely, point-of-care intervention when indicated [61].

The most common prescribing errors are incorrect drug, incorrect dose, allergies, and drug-drug interaction. Physicians have to keep the most common mistakes in mind and frequently check for errors.

Prior to prescribing any medication, the health-care professional must choose the appropriate medication for a given situation, considering factors such as allergies, route, dose, time, and regimen. Each patient may need a different treatment plan. It is important to tailor prescriptions for individual patients, identifying allergies, pregnancy, lactation, age, co-morbidities, breastfeeding, size, and patient weight. Health-care workers must be familiar with the medications they prescribe and need to know the medications in their specialty that are associated with high risk of adverse events. Remember the five R’s when prescribing and administering medication. Health-care professionals must monitor whether prescribed medication is clinically successful, does not cause harm, and is corrected when necessary.

Drug interactions can lead to serious adverse events or decrease drug efficacy [62]. Prescribing health-care workers should ask patients of any use of over-the-counter medications or dietary supplements because they are frequently under reported and may cause drug interactions [9]. Prescribing the generic name of drugs simplifies the communication among health-care workers, reducing errors. However, patients need to be educated that their medication may be called by different names (brand and generic name) and they should be encouraged to keep a list of their medications, including both the brand and generic name of each drug.

### Education and training medical students and surgical specialty residents

Technological advances, novel surgical devices, and minimally invasive techniques are rapidly increasing within the surgical community. Concerns about device safety and training are increasing, protecting patients from harm. Devices need to be extensively evaluated in research before and after FDA approval [63–67].

In teaching institutions, the participation of residents and fellows during the surgical procedure is integral to instill patient safety fundamentals in the trainees. Although involving trainees increases the duration of the procedure and increases length of stay for the patient, there are no significant differences in outcomes when trainees are involved. Residents and fellows provide extra assistance while also strengthening their skills to become knowledgeable and confident doctors [68–71].

Finally, due to new mandatory work hours residents are subjected to follow a pre-planned schedule during their duty hours and it is imperative to consider ensuring that patient safety is not compromised by breaks in the continuity of care. The handover process is a necessary bridge to continuity and safer patient care. Medical educators and clinicians should work toward adopting and testing principles of optimal handovers processes in their local practices applying the knowledge of patient safety issues discussed in this report [72].

## Conclusion

Patient safety is a significant issue within health care systems worldwide. Currently, guidelines for patient safety in surgery are general and not specific for each surgical subspecialties and training programs. The surgical environment must be considered as HRO, therefore, it demands a high level of standardization and safety processes in place with redundant system to decrease errors and human mistakes. Success in patient safety depends on several factors that include identification, revision of systems, education, and training to address known patient safety issues. Medical educators and mentors must understand and practice the culture of patient safety so the new generation of surgeons will incorporate the same values intuitively by mimicking the leadership.
